# Methodological framework for a user-centered, structured, and digitized training program for exoskeleton pilots in CYBATHLON preparation

**DOI:** 10.1186/s12984-026-01965-0

**Published:** 2026-04-19

**Authors:** Nicola Dobler, Christina Ortelt, Tobias Rieger, Lukas Schneidewind

**Affiliations:** 1 Medical Engineering, Technische Universität Berlin, Dovestrasse 6, 10587 Berlin, Germany; 2 Psychology of Action and Automation, Technische Universität Berlin, Marchstrasse 23, 10587 Berlin, Germany

**Keywords:** CYBATHLON, Exoskeleton training, User-centered design, Trainer qualification, E-learning, Assistive technology, Spinal cord injury, Methodology

## Abstract

**Purpose:**

Successful participation in the CYBATHLON depends not only on technical innovation but also on effective, systematic training of pilots with spinal cord injury. Current research primarily focuses on clinical rehabilitation, while standardized, task-specific training protocols for applied competition settings are lacking. To address this gap, this methodology paper presents a scientifically grounded training concept for exoskeletons, focusing on the qualification of trainers as key facilitators who adapt exercises and mediate between human and machine.

**Results:**

A modular 12-week training program with 38 units was developed and implemented through a Moodle-based e-learning platform, demonstrating the practical applicability of the proposed methodological framework. The program progresses from basic movement exercises to competition-specific obstacle tasks from the CYBATHLON Exoskeleton Race. The curriculum integrates theoretical content on safety and exoskeleton handling with practical training scenarios. Trainer qualification forms the central element, enabling individualized adaptation of exercises to pilot capabilities and exoskeleton requirements. The concept allows for scalable application and provides a consistent framework that can be updated according to future CYBATHLON regulations and technological developments.

**Conclusion:**

The developed user-centered and digitized training program provides an effective structure for preparing trainers and pilots for the CYBATHLON. Combining modular design, digital learning tools, and individualizable adaptation, it establishes a transferable framework applicable to other exoskeleton systems. Beyond its practical use, the framework introduces a replicable methodology for developing and implementing training concepts in human–machine interaction, forming a foundation for evidence-based and transferable training standards in assistive robotics.

## Background

### Introduction

In 2021, an estimated 574,502 new cases of spinal cord injury (SCI) were reported worldwide. Furthermore, the number is expected to be around 12 million people worldwide living with a spinal cord injury by 2050 [1]. SCI often occurs in young adults and leads to profound health and social consequences, most notably paralysis of the lower limbs, restricted mobility, and a wide range of secondary complications such as skin disorders, pain, spasticity, urinary tract infections, impaired cardiovascular capacity, reduced bone mineral density, and depression [2, 3]. Regaining walking ability is therefore a central rehabilitation goal for people with SCI, regardless of age, injury severity, or time since onset [4].

Robotic exoskeletons are externally worn, powered orthoses that support human movement or posture, and for the lower limbs, they enable standing, walking, and stair climbing through motorized assistance at the hip and knee joints [5]. Beyond mobility, exoskeleton-assisted training improves muscle activity, cardiovascular health, circulation, bowel and bladder function, and psychological well-being, while also reducing spasticity, pressure ulcers, and osteoporosis [6, 7].

Existing studies demonstrate functional gains from exoskeleton training, including increased walking distance, speed, and independence, as well as improved muscle strength and, in some cases, neurological recovery [8, 9]. However, standardized protocols for optimal training are lacking, as existing research has primarily focused on clinical rehabilitation. Establishing such protocols is essential to ensure reproducibility and effective knowledge transfer between research and applied practice. Task-specific training programs for applied contexts, such as the international CYBATHLON [10] competition where individuals with disabilities compete using assistive technologies, remain largely unexplored. Moreover, device-specific properties further hinder the development of universal training concepts.

To address these limitations, the presented work aims to develop a structured, user-centered training framework for exoskeleton pilots, specifically adapted to the requirements of the CYBATHLON 2024 competition [10], thereby linking rehabilitation-oriented research with task-oriented performance under competitive conditions. Its emphasis lies not on the direct training of pilots, but on the teaching of trainers as key actors in the implementation of the training process. Trainers may come from diverse professional backgrounds, though foundational physiotherapeutic understanding, including movement analysis and spinal cord injury-specific considerations, is beneficial. Any gaps in fundamental knowledge should be addressed through targeted continuing education or consultation with relevant experts while implementing this methodology. The aim is to provide trainers with the necessary knowledge and practical skills to select appropriate exercises based on individual pilot requirements and to instruct them while systematically considering device-specific constraints and safety-related requirements.

### Motivation and context

**Fig. 1:**
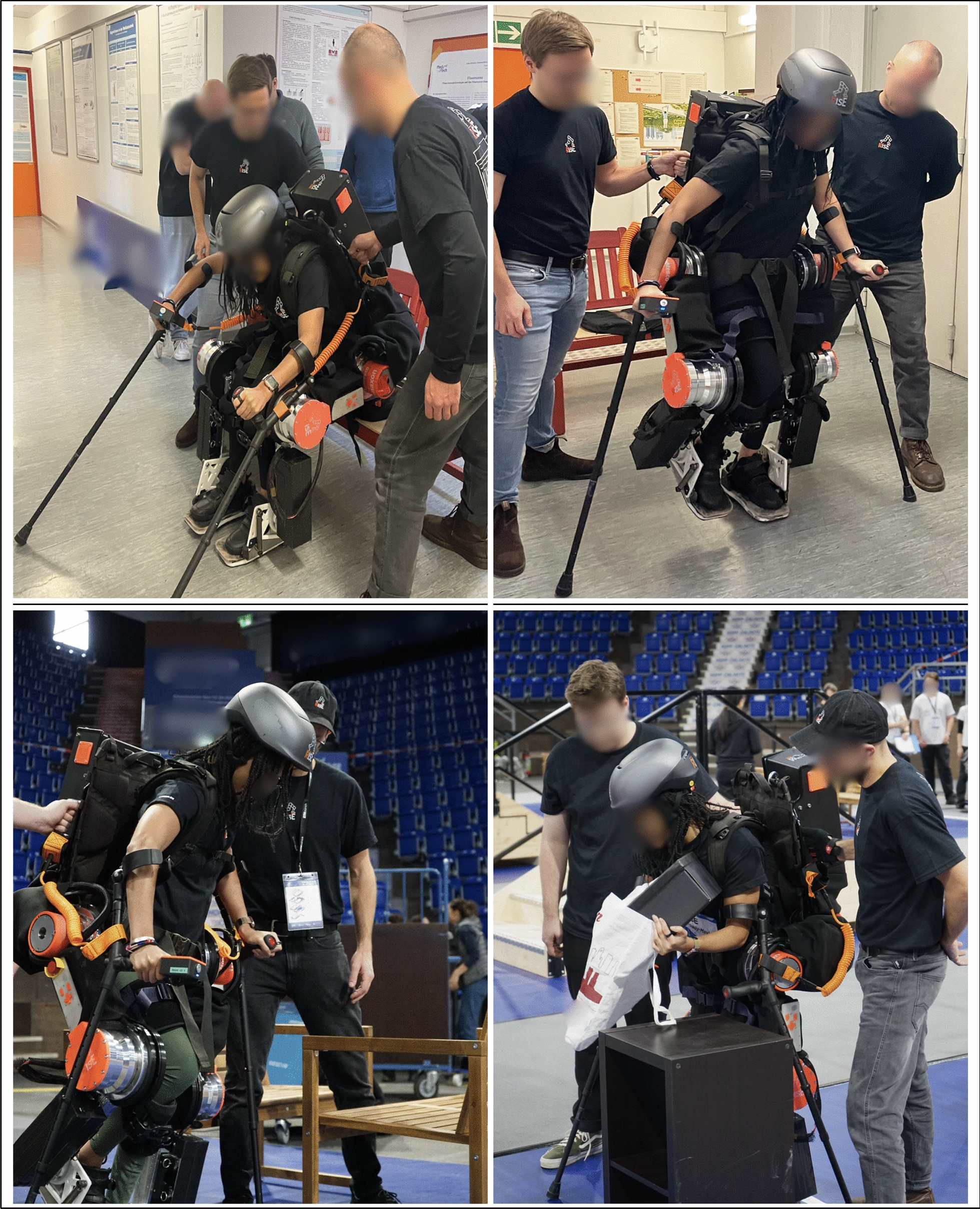
Pilot training sessions (top) and rehearsal training at CYBATHLON 2024 (bottom), illustrating the practical context for systematic training framework development

The presented work was developed in the context of the RISE project at Technische Universität Berlin, launched in October 2022, within which the authors participated with the exoskeleton RISE EXO I in the CYBATHLON 2024 competition. During training and the CYBATHLON competition in October 2024 (Fig. 1), it was observed that teams employed heterogeneous strategies for obstacle negotiation and that several demanding obstacles were not attempted by any team, such as *Stairs*, *Stony Path*, *High Step* and *Tilted Path* [10].

Discussions with team leads of competing EXO teams further revealed that training preparation for the competition varied substantially between teams, ranging from highly individualized routines to largely unstructured training phases. These observations indicated that preparation strategies were often selective and strongly dependent on individual team experience rather than on structured training concepts. Although individual teams have reported internal training protocols, no generalizable framework for preparing pilots and trainers for the specific task demands of the CYBATHLON could be identified, either in practice or in the existing literature. Moreover, no closely related training methodologies were found that could be readily adapted to the specific requirements of the competition. These practical experiences motivated the development of a user-centered training framework that enables exoskeleton teams to systematically design and adapt training programs tailored to specific competition requirements, individual pilot capabilities and device-specific constraints. The framework is intended to guide the creation of adaptable training programs rather than to define fixed training protocols, thereby supporting future CYBATHLON teams in building their own training concepts in a structured and reproducible manner.

### Discipline EXO at CYBATHLON 2024

**Fig. 2:**
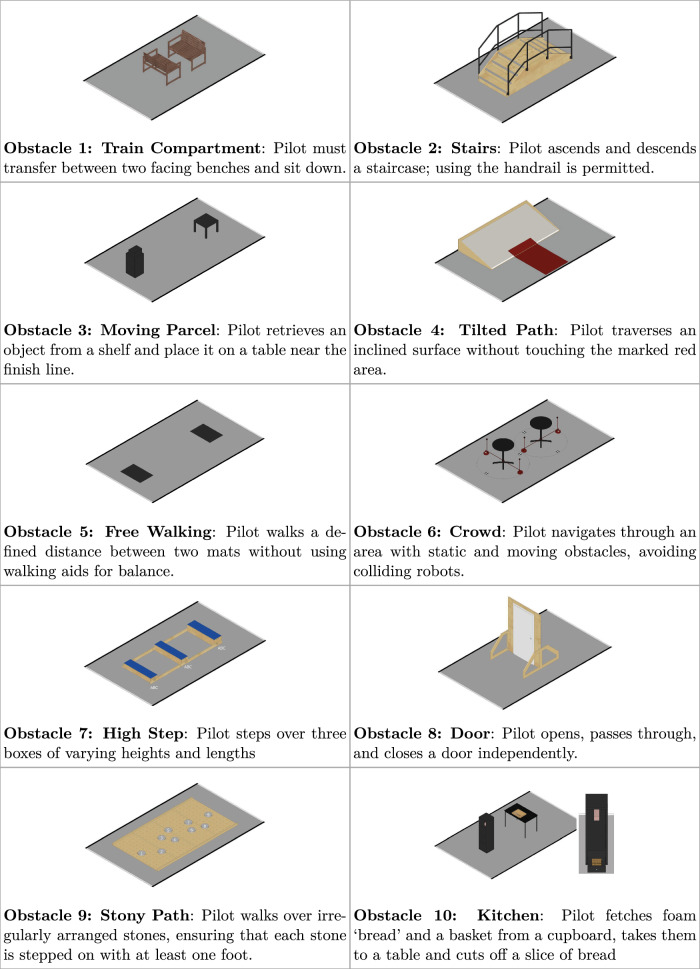
Overview obstacles CYBATHLON 2024 based on figures from ETH Zurich [10]

At the CYBATHLON 2024, the EXO discipline consisted of ten distinct obstacles, each designed to simulate everyday mobility challenges shown in Fig. 2. All tasks must be completed independently by each pilot and in the prescribed order. Wearing a helmet is mandatory, and walking aids must be carried throughout the entire race. Direct assistance or external control is not permitted.

## Methods

### Background analysis of relevant information

The methodology began with the systematic collection of information required for the design of an exoskeleton training program. Following an iterative principle, the process moved from a broad review of rehabilitation and assistive systems toward practice-oriented and device-specific content.

**Fig. 3:**
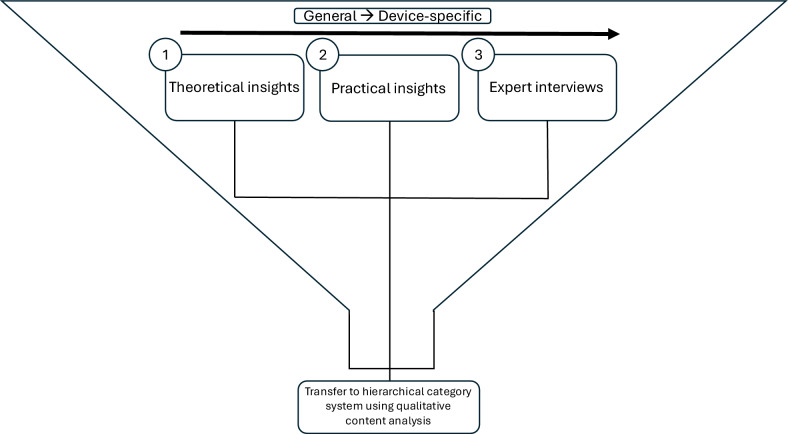
Structured procedure for literature and context analysis

In accordance with the procedure shown in Fig. 3, theoretical findings were obtained first. The selection of relevant studies was guided by defined inclusion criteria for CYBATHLON pilots [10]. Search terms included *exoskeleton*, *training*, *spinal cord injury*, and parapleg*, refined using Boolean operators AND/OR. Studies were excluded if they examined patients without complete motor loss of the lower extremities (e.g., multiple sclerosis, Parkinson’s disease, stroke) or focused solely on general health improvements without addressing exoskeleton training. Given the limited number of studies directly addressing exoskeleton training design, methodological approaches, structural principles, and training protocols were also analyzed. Particular attention was paid to how training with persons with paraplegia was conducted, even when it was not the primary research objective but rather a means to pursue other study goals.

Practical information was obtained through the analysis of publicly available video material depicting the use of various exoskeletons in rehabilitation contexts and a workshop conducted with a company in the field of exoskeleton manufacturing, which has an exoskeleton listed in the German medical aid register, on July 29, 2024, featuring a live demonstration with its device. In addition, four semi-structured interviews were conducted: three with experts in exoskeleton training to gain insights into existing training concepts, and one with a technical specialist to identify specific system-related requirements of the RISE EXO I, which served as an example case.

All theoretical, practice-oriented, and interview data were subsequently organized within a hierarchical category system. To systematically categorize the relevant content, a qualitative content analysis following Mayring’s approach was applied [11]. Core components were derived deductively from existing literature and inductively from the interviews. This combination allowed for the integration of established exoskeleton training requirements with practice-based insights. The resulting category systems were hierarchically structured, with overarching main categories subdivided into more specific subcategories. Each category represented an essential component of the overall training concept. To minimize content overlap, categories were defined and formulated from the trainers’ perspective, reflecting their role as the target users of the training concept.

The study-based analysis of exoskeleton applications revealed five key factors that are crucial for effective and safe training.

First, practice parameters and learning conditions form the theoretical foundation of effective training. The general literature on therapy and assistive systems emphasizes the importance of practice parameters such as frequency, duration, motivation, and feedback [12–15]. Similarly, practice conditions such as rest intervals, variability, and transfer are considered key factors influencing learning success [12, 16–18].

Second, foundational understanding and device familiarization are essential. Trainers should develop a comprehensive understanding of the exoskeleton to foster the formation of an accurate mental model and ensure safe operation [19, 20]. In parallel, a gradual familiarization process at the beginning of training helps reduce initial uncertainty and skepticism among pilots while enhancing motivation and confidence [21, 22].

Third, proper individualization and adjustment of the device play a decisive role. Precise adaptation of the exoskeleton to anthropometric characteristics such as body height, limb length, and hip width is crucial for training success [6, 20, 23, 24].

Fourth, risk management and injury prevention must be carefully considered. The most common risks include technical failures, user errors due to insufficient training, uncontrolled movements, and physiological incidents such as sudden drops in blood pressure [25]. Central preventive measures encompass structured training and clear instructions, the use of aids such as walkers or forearm crutches, close supervision and spotting of pilots, technical safety systems such as overhead tethering, regular equipment maintenance, and mechanical limits within the exoskeleton to prevent uncontrolled movements [19, 25–29]. Additionally, skin protection and comfort are critical aspects of safety management. Skin irritations and pressure sores, often resulting from improperly fitted systems, are among the most common complications [20, 25, 28, 30]. Regular inspection of contact areas, targeted pressure relief, adequate padding, and proper skin care are therefore essential preventive measures [20, 23, 26, 28, 30–33].

Fifth, and finally, structured training protocols and evaluation methods ensure systematic progression. Standardized protocols with clearly defined goals are recommended [26]. Training progress is commonly evaluated using standardized tests such as the 6-Minute Walk Test (6MWT) and the 10-Meter Walk Test (10MWT), which assess walking speed and endurance [34]. Subjective exertion is typically measured with the Borg scale, which rates perceived effort from 6 to 20 and correlates closely with heart rate [35]. Satisfaction and motivation are often captured through Likert-scale questionnaires or satisfaction surveys [24, 25], while objective assessments such as the Intermediate and Final Skills Tests provide structured evaluations of performance [33].

Furthermore, practical insights and interview analysis revealed the importance of organizational, technical, and team-related dimensions that are essential for exoskeleton training, which can also be summarized as five key factors.

First, the overall structure and duration of exoskeleton training differ across settings. Across all examined training settings, the total training duration ranged from approximately 9 weeks to 6 months, three training sessions per week with 30–45 min of active training time per session, depending on factors such as lesion level, prior experience, and physical condition of the pilot.

Second, appropriate preparation and post-session routines were identified as crucial for both safety and performance. Pilots were advised to wear tightly fitted, breathable clothes and compression layers to prevent friction and reduce the risk of skin irritation beneath contact areas. Mobility routines conducted before each session included targeted stretching and trunk-activation exercises to improve circulation and facilitate smoother donning of the exoskeleton. Standardized pre-session checklists ensured the correct alignment of fixation points and functional testing of the device before use, while post-session routines involved device inspection, documentation of technical parameters, and visual skin checks to identify potential pressure areas at an early stage. With regard to routines, the use of evaluation schemes based on points was also mentioned, which are used to assess individual exercises and thus make it possible to measure progress.

Third, the coordinated work of interdisciplinary teams proved essential for high-quality training outcomes. In these interdisciplinary teams, clearly defined roles, including trainers, spotters, technical specialists, medical staff, and documentation personnel, communicate transparently. Team continuity further strengthens mutual trust and supports a consistent learning atmosphere.

Fourth, meticulous technical preparation and the implementation of emergency procedures were indispensable components of safe training practice. Regular inspections, systematic battery management, and precise device initialization contributed to technical reliability. Observed emergency procedures demonstrated how practiced team responses, such as stabilizing the exoskeleton, positioning a chair, or conducting a controlled evacuation in case of system failure, minimize risks and reinforce pilot confidence. Learning emergency behavior, including the simulation of a gentle fall during which the pilot stabilizes themselves with crutches and transfers under supervision, was introduced from the first training session onward. It should be noted that the insights from the interview with the technical specialist refer to the RISE EXO I model. Key technical specifications of this model included the device weight (around 85 kg), a center of mass positioned posterior to the hip flexion extension joint axis during standing and consequently posterior to the pilot’s center of mass, and an emergency stop function that activates Safe Torque Off, eliminating actuator torque output and causing the system to collapse into a passive, unloaded state. The device was underactuated (6 active, 4 passive degrees of freedom) and operated in open loop without actuated hip rotation or balance control, requiring the user to maintain stability and execute turning maneuvers using crutches.

Finally, the analysis of movement practice and training content revealed that exoskeleton training focuses on fundamental patterns such as targeted weight shifting, controlled turning, and the double-step technique with forearm crutches. Each of these movements is supported by coordinated trainer assistance. The execution of these patterns requires a high level of coordination, accurate guidance of the crutches, and deliberate postural control to maintain balance throughout the movement sequence [36–38]. The analysis of sit-to-stand and stand-to-sit strategies revealed standardized procedures in which the placement of the forearm crutches and the synchronized movement of the trunk play a decisive role [39]. Furthermore, obstacle-specific movement strategies demonstrated the necessity of individual adjustments in step length, crutch positioning, and arm strength [40–43]. Observations from past CYBATHLON races further underlined the importance of creative, adaptive movement strategies that extend beyond standardized gait patterns and address the specific challenges posed by individual obstacles.

Overall, the analysis revealed the key factors essential for safe and effective exoskeleton training. These findings form the central foundation for structuring them into a consistent training concept and further developing them didactically.

### Four-component instructional design model

For the development of a theoretical training concept based on the collected and analyzed data, an adapted model was designed, derived from and conceptually aligned with van Merriënboer’s Four-Component Instructional Design (4C/ID) model [44].

**Fig. 4:**
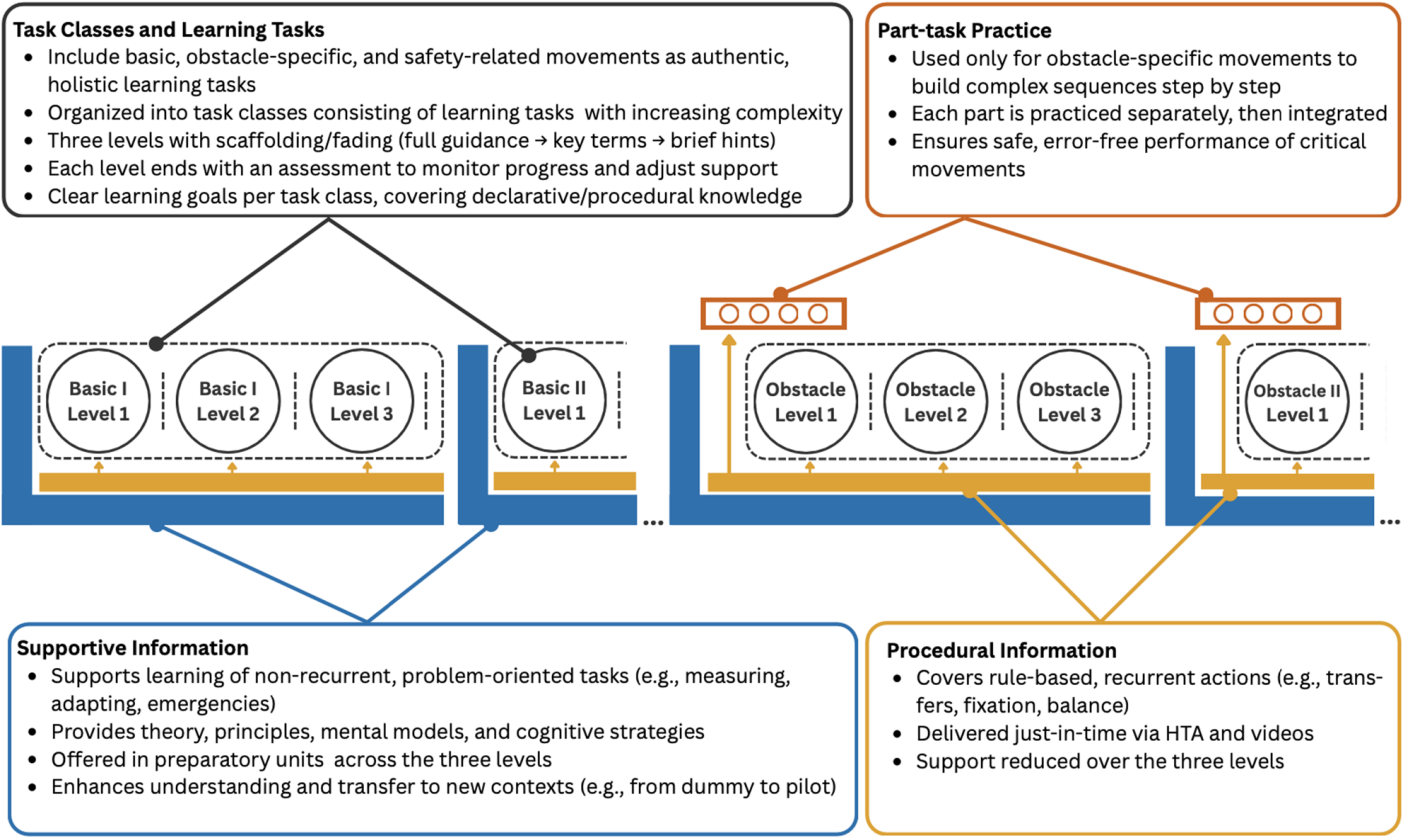
Adapted model for training concept development based on the Four-Component Instructional Design Model (4C/ID) by van Merriënboer [44]

The adapted 4C/ID model, shown in Fig. 4, provides a comprehensive framework for designing instruction that promotes the acquisition of complex skills for exoskeleton training. It comprises four interrelated components: Task Classes and Learning Tasks, Supportive Information, Procedural Information, and Part-task Practice. In this step, the skills to be developed are identified. A skill can be understood as performance in a specific task that develops through practice and draws upon task-independent, person-specific abilities [45]. Furthermore, the sequence of tasks within the modules is determined, the required materials are specified, and the methods for knowledge transfer and assessment of learning outcomes are defined [44].

#### Task classes and learning tasks

The learning tasks consist of authentic, practice-oriented activities that reflect the full scope of the skills to be acquired. They are holistic tasks based on real-world professional activities [44]. In this model, task classes comprise sets of learning tasks that aim to develop the same competencies. Within each class, instructional support is gradually reduced, represented by three levels. This reduction progresses from complete guidance (level 1) to keyword-based cues (level 2) and finally to brief reminders (level 3), thereby fostering independent execution and the automation of the required action sequences. Across classes, the complexity of the learning tasks rises progressively.

In this training concept, the classes of learning tasks correspond to the ten CYBATHLON obstacles, which together constitute the complex skills required for successful competition performance. However, the qualitative content analysis revealed that each obstacle comprises several basic skills that represent essential sub-competences of the overall task. To effectively master the obstacles, these basic skills must first be trained in isolation and are therefore defined as independent, subordinate classes of learning tasks in the subsequent design. In addition, the analysis identified safety-related activities such as measuring the pilot, adjusting walking aids, fixator fixation, emergency and fire safety procedures, safety check-ups, and balance exercises. These activities are essential for ensuring safe and efficient training implementation and are likewise integrated into the concept as distinct task classes. Accordingly, Table 1 presents the classes of learning tasks defined for the training concept.

**Table 1:** Overview of task classes categories and associated exercises

Safety-related task classes	Measuring the Pilot, Adjusting the Walking Aids, Fixator Fixation, Emergency Behavior, Fire Behavior, Safety Check-Up, Balance Exercise—Lifting the Walking Aids, Balance Exercise—Weight Shifting
Basic task classes	Transfer into the Exoskeleton, Transfer into the Wheelchair, Standing up (sit-to-stand), Sitting down (stand-to-sit), Standing up from the Trolley, Sitting down on the Trolley, Turning while standing, Double Step, Side Step, Standing Position
CYBATHLON-specific task classes	Train Compartment, Stairs, Moving Parcel, Tilted Path, Free Walking, Door, Kitchen, Stony Path, Crowd, High Step

Training begins with simple tasks, as complex and coordinatively demanding activities can only be effectively practiced once fundamental skills have been established. In the training concept, all basic movement patterns are considered basic task classes, whereas the CYBATHLON obstacles are classified as complex (Table 1). This implies that each training session first focuses on safety-related activities and fundamental movement patterns, followed by the CYBATHLON-specific obstacles that build upon these foundations. The sequence of safety-related skills and fundamental movement patterns follows a logical structure based on the prerequisites required for subsequent, more complex tasks and movements. At the beginning, safety-related skills must be taught to prevent injuries and accidents throughout the training process.

To classify the CYBATHLON obstacles according to levels of difficulty, a pairwise comparison method is employed to systematically determine their relative complexity [46]. The evaluation is based on the requirements identified during the background analysis of information, including strength, balance, safety, and the combination of fundamental movement patterns, as well as the expert assessment of the RISE project leader. The obstacles *Tilted Path* and *Free Walking* are not currently assigned to any difficulty level due to technical limitations of the EXO I exoskeleton mentioned in “Background analysis of relevant information” section. For the tasks *Moving Parcel*, *Door*, and *Train Compartment*, the official CYBATHLON 2024 assessment serves as an additional basis for classification. Based on this classification, Table 2 presents all task classes with increasing degree of difficulty.

**Table 2:** Ranking of safety-related, basic, and obstacle task classes with increasing degree of difficulty with classification into recurrent (rec) and non-recurrent (non-rec)

Task class	Rank
Safety-related/basic task classes	
Measuring the pilot (non-rec)	1
Adjusting the walking aids (non-rec)	2
Transfer into the exoskeleton (rec)	3
Fixator fixation (rec)	4
Transfer into the wheelchair (rec)	5
Safety check-up (rec)	6
Standing up (sit-to-stand) (rec)	7
Standing position (rec)	8
Emergency behavior (non-rec)	9
Fire behavior (non-rec)	10
Sitting down (stand-to-sit) (rec)	11
Sitting down on the trolley (rec)	12
Standing up from the trolley (rec)	13
Balance exercise—weight shifting (rec)	14
Balance exercise—lifting the walking aids (rec)	15
Turning while standing (rec)	16
Double step (rec)	17
Side step (rec)	18
CYBATHLON obstacles (rec)	
Moving parcel	19
Door	20
Train compartment	21
Crowd	22
Kitchen	23
Stony path	24
Stairs	25
High step	26

In addition to classifying the task classes by difficulty, it is essential to distinguish between recurrent and non-recurrent skills. Recurrent skills are based on rule-driven processes and are performed consistently after training through fixed sequences of actions. In contrast, non-recurrent skills rely on schema-driven processes that vary across problem situations. They require cognitive control and the application of problem-solving strategies [47].

This distinction is crucial for the design of the training program, as it determines whether supportive or procedural information is needed for each skill. Within the classification of tasks for this training, skills are considered recurrent when they rely on repetitive movement elements with rule-based action sequences, including safety check-ups, fixation procedures, and movement patterns such as transfers to and from the wheelchair, as these activities are performed according to fixed rules in every training session.

The non-recurrent tasks include measuring the pilot and adjusting the walking aids, as these activities are not performed routinely and their level of difficulty may vary depending on the individual’s physical characteristics. Factors such as differences in skin or fat layers can complicate the measurement process and require alternative approaches. Similarly, behavior in emergency and fire situations is classified as a non-recurrent task. Such situations do not follow standardized procedures but instead require adaptation to the specific circumstances. Emergency behavior during stair negotiation differs from that required when walking on level ground. In the event of a fire, context-dependent decisions must be made, such as whether the batteries can be safely disengaged or whether a tool should be used to knock them off. For these non-recurrent tasks, it is therefore essential to provide cognitive schemas that enable the training staff to develop appropriate strategies for effective and situation-specific responses.

The formulation of learning objectives for the previously identified tasks is essential for evaluating the trainers’ learning outcomes, as the acquired knowledge can be compared with the predefined criteria. Learning objectives are differentiated into general, intermediate, and specific objectives. General learning objectives define broad areas of competence or knowledge domains, while intermediate objectives describe overarching competencies. Specific learning objectives, in turn, operationalize these competencies at a more detailed level and are formulated for individual modules or instructional units [48]. In defining these objectives, emphasis is placed on clarity and informational precision [49]. Particular attention must be paid to ensuring that the objectives are formulated in a way that allows for systematic and transparent evaluation through measurable indicators, thereby enabling the comparison of achieved outcomes with predefined criteria and supporting the overall quality assurance of the educational process [50].

**Fig. 5:**
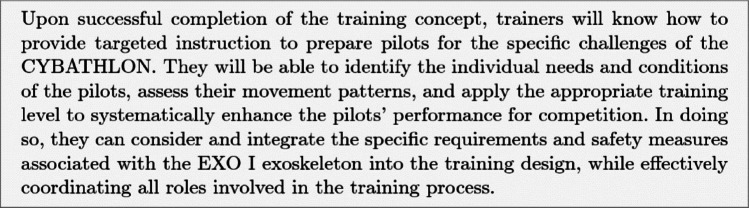
General learning objective for training concept

The learning objectives were formulated using active and precise verbs to ensure that they are observable, measurable, and assessable. The achievement of specific action-oriented goals often presupposes the attainment of corresponding knowledge-related objectives. For each learning objective, it was determined whether it pertains to declarative or procedural knowledge, as this distinction informs the selection of appropriate assessment methods: Declarative knowledge is evaluated through quizzes, while procedural knowledge is assessed by gradually reducing trainer guidance. Both forms of knowledge are present across all task classes, regardless of their classification as recurrent or non-recurrent.

The structure of intermediate and specific learning objectives is illustrated using two examples: *Emergency Behavior* (non-recurrent) and *Transfer into the Wheelchair* (recurrent). The example of emergency behavior illustrates the interplay between declarative and procedural knowledge. The intermediate objective is the safe and correct execution of schema-based responses in emergency situations, encompassing both the understanding of underlying rules and the practical performance of action sequences. The specific learning objectives comprise both the understanding of the underlying rules (declarative knowledge) and the practical execution of the action sequences (procedural knowledge). Materials such as schemas and behavioral guidelines are first provided to establish declarative knowledge, which is assessed through a quiz before practical application. The procedural learning objectives of non-recurrent tasks are evaluated through three levels of increasing difficulty, in which the provision of information essential for task execution is gradually reduced.

**Fig. 6:**
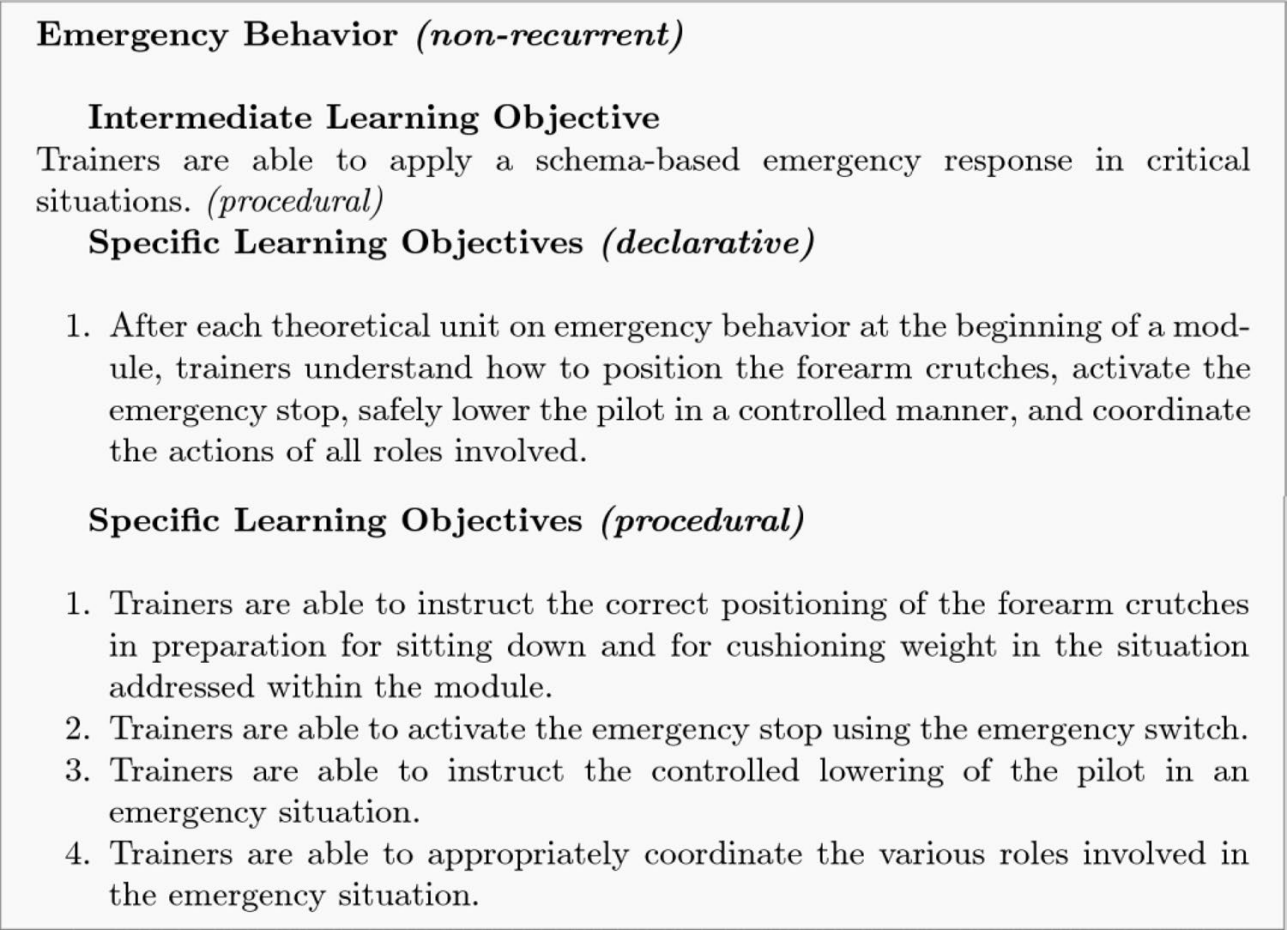
Intermediate and specific learning objectives of the Emergency Behavior

For the transfer into the wheelchair, the declarative learning objectives involve understanding theoretical aspects such as correct posture, movement sequences, the role of assisting personnel, and measures to prevent pressure sores and misalignments. The procedural learning objectives address the practical execution, including coordination of assistants, evaluation of movements, and adjustment of pressure points or joint positions. Declarative objectives are assessed by having trainers arrange the individual steps of the transfer in the correct order. Procedural objectives are evaluated by gradually reducing instructional support through three levels, starting with detailed video demonstrations at level 1, followed by key terms at level 2, and brief prompts at level 3 to foster autonomy and automatization of task performance.

**Fig. 7:**
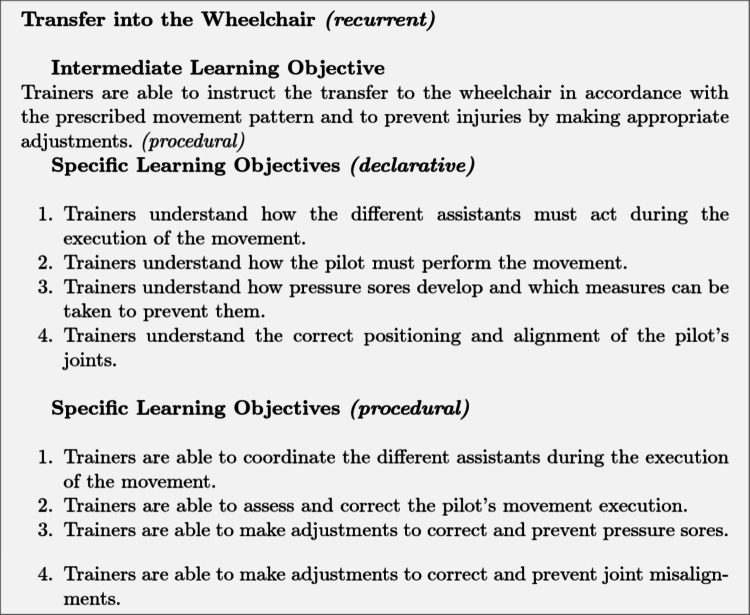
Intermediate and specific learning objectives of the Transfer into the Wheelchair

#### Supportive information

The supportive information comprises theoretical knowledge, principles, and cognitive strategies that are essential for addressing the non-recurrent, problem-solving aspects of the identified tasks. They assist learners in understanding the underlying concepts and are provided either prior to or during task execution [44]. In the training concept, supportive information is delivered through presentations, handouts, and structured exercise units. The aim is to foster a deep understanding of complex and variable situations and to enable the flexible application of acquired knowledge.

To this end, preparatory sessions are integrated into the training concept. The purpose of these sessions is to enable trainers to develop a mental model of the respective subject areas and tasks. This mental model facilitates the transfer of knowledge and skills to novel situations encountered later during practical training with the pilot. Moreover, the preparatory sessions incorporate a gradual reduction of supportive information, implemented through three difficulty levels.

In the first preparatory session, which focuses on *Measuring the pilot* and *Adjusting the walking aids*, the tasks are practiced across levels 1–3 with progressively decreasing amounts of supportive information. During these stages, measurement exercises are conducted on different individuals to promote flexibility and understanding.

In the second preparatory session, dedicated to *Emergency behavior*, the training is likewise structured in progressive levels. In level 1, the activation of the emergency stop system is practiced using an empty exoskeleton secured by a safety rope–first with a seat, then without. In level 2, the training is performed without the safety rope, starting again with the empty exoskeleton and seat before progressing to exercises without the seat. In level 3, the emergency procedure is practiced with a dummy fixed inside the exoskeleton, also without rope safety–first with a seat, then without a seat. Finally, the trained action schemas are reflected upon collectively with all involved roles, and situation-specific aspects are discussed to ensure safe application in real emergencies.

The third preparatory session addresses *Fire behavior* and is conducted solely on a theoretical level. Practical exercises are omitted, as the physical removal of batteries could potentially damage both the exoskeleton and the batteries themselves. Therefore, a theoretical action schema is provided to convey this non-recurrent skill.

The fourth session serves as an introductory meeting involving all roles, including the pilot. During this session, the pilot is introduced to the exoskeleton, the CYBATHLON, and the various training roles. The objective of this initial meeting is to familiarize the pilot with the training environment and establish mutual trust. Subsequently, the pilot’s measurements and the adjustment of the forearm crutches are carried out using the supportive information provided.

A distinctive feature of the training modules involving the pilot is that any training content previously practiced with other individuals or a dummy begins at level 2 when transferred to the pilot context. This ensures an appropriately adjusted level of difficulty and facilitates the transfer of skills to the individualized interaction with the pilot.

#### Procedural information

The procedural information provides specific guidance for recurrent, routine skill components that follow fixed rules. This information specifies precisely how these routines are to be performed and is provided only when needed. As learners gain experience, the amount of procedural information is gradually reduced until it is no longer required [44].

A methodological approach for the structured decomposition of complex action sequences is described by Barfield [51] within the framework of Hierarchical Task Analysis (HTA). In this approach, overarching tasks are divided into a hierarchical sequence of subtasks, which are further broken down into the smallest meaningful steps. This method helps to reduce the complexity of demanding action sequences in exoskeleton training and provides a consistent and reproducible structure for recurrent movements. Particular attention was paid to ensuring that the HTAs are formulated with precision. They are presented either as instructional guides that the trainer communicates to the pilot or as specific directives for the trainer, highlighting task-relevant aspects that must be observed. Within the individual HTAs, certain fundamental skills, such as the double step or standing position, are referenced multiple times, as they represent essential components of various movement sequences. In this way, individual skills are interconnected, as basic movement patterns and safety check-ups are reused across multiple higher-level procedures.

The provision of procedural information takes place deliberately at the beginning of each learning unit. This follows the just-in-time principle, ensuring that learners receive the necessary information precisely when it is required for the successful execution of task steps [44]. The procedural information is conveyed through detailed instructional videos that illustrate the HTA and the corresponding sequence of actions. Trainers must internalize these sequences independently before applying them with the pilot. The correct application of procedural information requires specific prior knowledge. The corresponding action sequence, presented in the form of HTAs, is considered prerequisite knowledge that enables the correct implementation of procedural information. This prior knowledge is assessed through quizzes in which trainers are required to arrange the HTA steps in the correct order before applying the procedures during practical training.

As outlined in the development of the supportive information, the procedural information is also provided in three successive levels of support to promote the gradual reduction of guidance and the automation of trainers’ skills. After completing all three levels, trainers should be able to instruct movements and exercises with increasing automaticity, without relying on the HTAs as support. In level 1, the complete HTA is provided, allowing trainers to understand the entire sequence of actions. In level 2, the support is reduced to key terms representing the main steps of the HTA. In level 3, only brief prompts are given to encourage independent and automated execution of the training instructions.

Furthermore, a fixed sequence has been defined for all fundamental recurrent tasks, as these build upon one another. Sitting down, for instance, must be practiced after standing up, and the double step is introduced only after balance control has been developed. Similarly, in obstacle-specific training, individual movements are interdependent and should therefore be practiced progressively and in a fixed order from start to finish, rather than in randomized sequences.

#### Part-task practice

The part-task practice consists of specialized exercises designed for recurrent or safety-critical skills that require a high degree of automation. Part-task practice is implemented only after the skill has been introduced within a holistic task. Its purpose is to enable the automated execution of routine action sequences [44].

Due to the complexity of the CYBATHLON obstacle-specific tasks and the potential safety-critical consequences of errors, each obstacle is divided into individual part-tasks. Performing the entire sequence of movements immediately would not be appropriate given the associated safety risks. It is therefore essential that trainers first internalize the complete HTA and the corresponding instructional video for obstacle negotiation to ensure optimal preparation.

Subsequently, the movement sequence should be taught step by step through the structured instruction of part-tasks. This approach prevents the pilot from becoming overwhelmed and ensures that each component of the movement sequence is mastered correctly before executing the entire task.

With regard to the implementation, the first part-task is trained in isolation. The second part-task is then appended to the first so that both are practiced as a contiguous sequence. This process is continued step by step until the entire sequence of movements is mastered as a complete chain. The order of part-tasks follows a fixed sequence to ensure the correct progression of movements for each obstacle. Successful execution of a later movement depends directly on the successful performance of the preceding segments. For example, descending stairs cannot be practiced in isolation before the ascent has been completed.

### Development of the training program structure

To systematically develop the temporal structure of the training program, existing training schedules from other CYBATHLON teams were collected and analyzed to identify common patterns in timing, frequency, and module sequencing. An overview of the analyzed training programs is provided in Table 3.

**Table 3:** Overview of analyzed training programs listed by code of the interviewee

Source (interviews)	Sessions (count)	Duration (min)	Frequency (count)	Weeks (count)	Active (min)
Clinical Training Manager	36–58	75	3	12–24	35–40
P2_Cybathlon Team 1	36–48	240–360	3	12–16	30
P3_Cybathlon Team 2	58	90	3	24	45
Average	43–54	135–175	3	16–21.3	36.7–38.3

The final training plan for the EXO I exoskeleton extends over a period of twelve weeks. The first five weeks are dedicated to the performance of fundamental exercises, followed by obstacle-specific training to prepare for the competition. The training is conducted at a frequency of three sessions per week, resulting in a total of 38 training sessions that build systematically upon one another. This structure enables a continuous increase in task complexity and a gradual reduction of external support.

The temporal structure of the developed training plan shown in Fig. 5 represents an idealized minimum program and lies below the average training duration identified. This provides sufficient flexibility to accommodate realistic training conditions, including longer practice phases, adaptation loops, or the repetition of specific sessions if necessary.

**Fig. 8:**
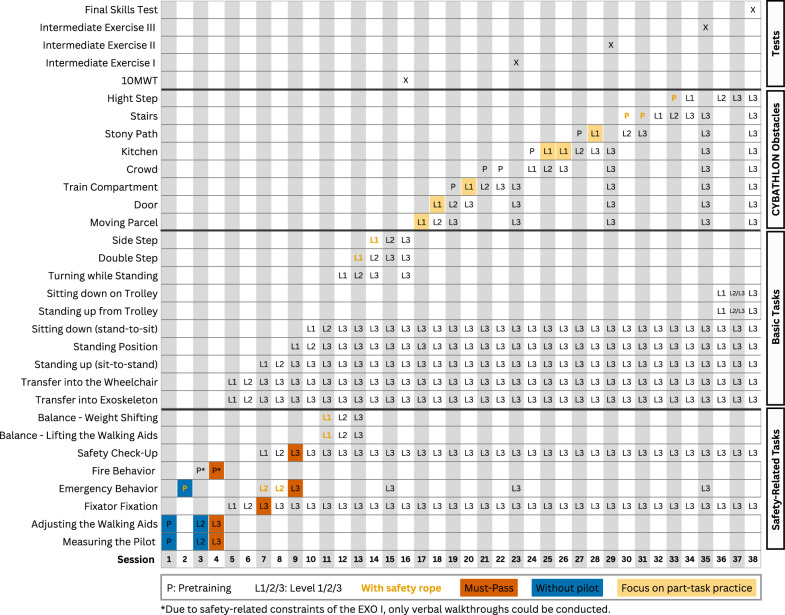
12-week training schedule with 38 sessions, progressing from fundamental exercises to obstacle-specific tasks, allowing gradual increase in complexity and reduction of support

### Digital implementation

The digital training module was implemented on a digital learning platform of Technische Universität Berlin. As an established, university-wide e-learning environment, it is based on the open-source system moodle. The training was designed as a self-directed and self-explanatory learning environment that requires no additional external supervision. A key advantage of the platform lies in its ability to control learning progress through the configuration of access conditions. This mechanism guarantees that trainers first understand and internalize all relevant content before it is applied in practice, thereby enhancing safety during pilot sessions.

#### Acknowledging human limitations: cognitive load theory

The design of cognitively efficient learning materials in this study is based on Cognitive Load Theory according to Sweller et al. [52], which distinguishes three types of cognitive load: Intrinsic, extraneous, and germane. These three forms together determine the total load imposed on working memory, whose processing capacity is inherently limited. Intrinsic cognitive load arises from the inherent complexity of the learning content and the interactivity of its elements, but it can be managed through instructional strategies such as segmenting complex information and providing relevant prior knowledge. Extraneous cognitive load, by contrast, is influenced by the design of the learning materials and should be minimized by avoiding redundant information and distracting elements. Germane cognitive load refers to the mental effort invested in the deep processing of information, occurring when learners integrate new content into existing knowledge structures and refine their cognitive schemas.

#### Trainee-friendly training materials: principles of multimedia design

Building on the Cognitive Load Theory, the design of the training materials systematically addresses the three types of cognitive load. The following principles of multimedia design, developed by Clark and Mayer [53], are grounded in theories of human learning and in extensive empirical evidence. Applying these scientifically validated principles enables a reduction in extraneous cognitive load and allows for the deliberate management of intrinsic load. At the same time, this approach maximizes the cognitive capacity available for germane processing, thereby fostering optimal conditions for effective and sustainable learning.

These principles serve as practice-oriented guidelines for aligning the design of the training materials with the cognitive processes involved in human information processing.

**The pretraining principle**

According to this principle, learning becomes more effective when essential terms and concepts are introduced prior to the main instruction. In line with the pretraining principle, these key elements are presented to the trainees in advance to build a foundational understanding of concepts and procedures [53].

On the course homepage, trainers are first greeted with a welcome message that outlines the purpose and objectives of the program. This is followed by introductory information that provides guidance on how to get started and outlines the essential information trainers need at the beginning of the course. Before starting with session 1, trainers are instructed to complete all materials provided under the tab *General information*. Moreover, corresponding quizzes have to be passed, as these form the foundation for a safe and successful entry into the training program. Subsequently, the contents provided under the tab *General information* and their relevance to the overall training process are outlined, offering an overview of the materials and how they support the trainers throughout the course. It includes a glossary providing definitions of essential terminology used in the training program, an overview of the training sessions (Fig. 5), templates for the training session (Appendix C) and training log, as well as preparation and follow-up checklists (Appendices A, B), evaluation schemes (Tables 8, 9), an example training plan, and preparation materials (Appendix E, Fig. 6) for the CYBATHLON competition, the RISE project, the exoskeleton and the respective roles involved in the training program.

The *structural and temporal organization* of the training program is explained in the following section, outlining the division into sessions and modules as well as the use of levels within the training process. Furthermore, criteria for successful session completion are also specified.

For organizational purposes and to facilitate material reuse, the final section of the introduction provides guidance on the *file collection for editing tab*. This section offers editable versions of course materials for individual adaptation and includes a *lessons learned* area designed to document general insights and reflections from the training process.

**The segmenting principle**

The segmenting principle is implemented by dividing the training into small, logically sequenced units to reduce cognitive load [53]. The introductory section, *General information*, provides all templates, introductory materials as well as schedules and should be completed before the start of the actual training.

The context-specific structure of the course is organized into three main sections, under which the respective modules are grouped: Modules 1–5 cover the safety-critical introduction, modules 6–16 focus on basic movements, and modules 17–27 address CYBATHLON-specific movements. Each of these three sections is presented under a dedicated tab, with additional sub-tabs for the individual modules contained within it. At the start of each of the three main sections, introductory information provides essential context about that part of the training, covering its relevance, practical guidance for implementation and an overview of the modules included in the section.

The temporal structure of the training itself consists of 38 sessions distributed over a twelve-week period. Each session is linked to the corresponding tab that contains the respective thematic module. Every training module covers either a fundamental movement pattern or a specific obstacle task from the CYBATHLON and follows a consistent instructional structure. Depending on the type of skill (recurrent or non-recurrent), the module begins with an introductory video including a HTA or a presentation with supplementary information. This is followed by a short quiz to verify understanding and a series of practical exercises organized into three progressive levels of difficulty.

**The personalization principle**

According to the personalization principle, a personalized style of communication enhances learners’ motivation and engagement [53]. To apply this principle in the training program, a direct and respectful second-person form of address is used. This is complemented by the choice of a natural human voice to create an authentic and accessible learning environment.

The visual design aligns with the color scheme and design elements of the RISE platform, ensuring a consistent user experience that supports orientation and recognition across modules. The combination of verbal, auditory, and visual elements aims to foster learner involvement and promote a supportive and engaging learning atmosphere.

**The multimedia principle**

The multimedia principle states that combining text and visuals leads to better understanding than presenting text alone [53]. Therefore within the training platform, visuals are purposefully combined to make abstract information more tangible and to facilitate comprehension of the content. For each HTA, a corresponding video is provided that demonstrates the execution of the movement. Subtitles are displayed alongside the spoken narration, enabling individuals with hearing disabilities to fully access the content. A sample screenshot from the implementation of these videos can be found in Appendix F, Fig. 7. Since many movements cannot yet be technically performed with the exoskeleton, the demonstrations are shown without the device but follow the actual movement patterns of the system. The correct use of the forearm crutches and the strategic approach to each obstacle are emphasized.

**The coherence principle**

The coherence principle is implemented through the deliberate exclusion of distracting content to focus the learning process [53]. All graphics, audio elements, and texts are functionally integrated and serve exclusively to support the respective learning objectives. Decorative images or background music were intentionally avoided to minimize cognitive load and keep the learners’ attention focused on the essential information. This reduction to the essentials creates a focused learning environment that efficiently supports knowledge acquisition.

**The contiguity principle**

Learning is supported when related text and images are presented in close spatial and temporal proximity [53]. The training platform addresses this principle by providing direct labels for relevant components within the graphics. Figures, such as the one showing the fixators and casing of EXO I (Appendix E, Fig. 6), employ arrows to visually highlight and label the relevant components, ensuring that their function and position can be easily identified. In the videos, temporal contiguity is ensured by synchronizing the narrated Hierarchical Task Analysis with the corresponding movement execution. This narration also demonstrates the application of the modality principle, as it reduces visual channel overload and allows learners to process verbal and visual information in parallel without the need to alternate between written text and imagery.

### Summative evaluation prior to practical training period

As a recommended supplement to the methodology’s implementation, summative evaluation with potential trainers before practical application of the developed training program can enhance user-centeredness and verify comprehensibility. In addition, it may identify potential ambiguities that may not be apparent during the practical training process.

For the implemented digital training module, an evaluation was conducted through a focus group with five participant who served as trainers in preparation for CYBATHLON 2024, providing them with relevant prior experience to assess the module. The participants were provided access to the complete digital training platform and instructed to work through the materials independently during a self-study phase. Subsequently, a moderated group discussion was facilitated to systematically gather feedback on structural clarity, terminological precision, practical applicability, and perceived barriers to implementation. Supplementary written feedback forms can capture individual perspectives that did not emerge during group discussion.

The collected feedback was categorized into immediately actionable modifications as well as prospective enhancements requiring further consideration. Actionable feedback was implemented directly to optimize the training program before practical deployment. This approach of user-oriented refinement minimizes potential barriers arising during the training phase and ensures that the training materials are tailored to the practical needs of future key users, ultimately maximizing the applicability of the methodology in practice.

## Results

A complete version of the final training plan, including the detailed temporal distribution of the twelve-week program, is presented in Fig. 5 in “Development of the training program structure” section. In the following section, an exemplary module is described to exemplify the overall structural layout, instructional methodology, evaluation procedures, and progression strategy implemented across the entire training program.

Module 17 *Moving Parcel* is used as an example to illustrate the structure of the learning content, the methodological sequence and the implementation of assessment mechanisms. This module was selected because, at this point in the training schedule, task variability is still low since no other Cybathlon obstacles have been trained yet. It therefore clearly illustrates the structure of a training session and the progression of learning tasks. Table 4 provides the temporal schedule of session 17, which aims to enable the acquisition of the *Moving Parcel* obstacle at level 1. The session plan provides step-by-step guidance for the trainer, specifying which materials are to be used at each stage of the session. Module 17 within the digital learning course therefore contains all materials required for conducting Session 17 during the pilot training. Appendix D shows module 17 on the e-learning platform.

**Table 4:** Training schedule for session 17: *moving parcel*

Training step	Min	Content/activity
Preparation (without pilot)	–	Check safety instructions, watch video, complete quiz in Module 17
Start	15	Welcome and arrival of all participants
Preparation checklist	15	Fill in the preparation checklist for this session
Setup	15	Transfer, fixators, safety check-up, standing up
Moving parcel (task 1)	20	Module 17: moving parcel (level 1)—task 1
Interim evaluation 1	5	Evaluate task 1 (pilot/trainer, minimum 4/5 points)
Moving parcel (task 1 + 2)	20	Module 17: moving parcel (level 1)—task 1 + 2
Interim evaluation 2	5	Evaluate task 1 + 2 (pilot/trainer, minimum 4/5 points)
Break	10	Rest, check for pressure points, remove exoskeleton if needed
Moving parcel (task 1 + 2 + 3)	20	Module 17: moving parcel (level 1)—task 1 + 2 + 3
Interim evaluation 3	5	Evaluate task 1 + 2 + 3 (pilot/trainer, minimum 4/5 points)
Wheelchair transfer	5	Module 7: transfer into the wheelchair
Debriefing	15	Complete debriefing checklist, group reflection, check definition of done

Each training session has a total duration of approximately 150 min and includes preparatory and debriefing phases of about 15 min each. Regular breaks are an integral part of the structure: In particular, after training phases during which the pilot remains in an upright standing position for at least 35 min, a recovery period is mandatory. These breaks serve to prevent overuse, avoid pressure-related discomfort, and maintain performance capacity throughout the entire session.

### Preparation without the pilot

Before the start of each new training session involving specific requirements related to the non-recurrent skills of emergency and fire behavior, the trainer receives renewed instructional guidance, displayed at the beginning of the corresponding module. This repetition serves to reactivate previously acquired knowledge and ensures that the required behavioral responses can be correctly and appropriately executed in case of an emergency. If an unexpected emergency or fire situation occurs during standing up or any other movement, the trainer must decide, based on the situation, whether the emergency procedure should be performed with or without a seating aid. This decision depends on the immediate situational assessment and the available reaction time.

Correct behavioral execution in all conceivable scenarios must be fully internalized and retrievable by all participants involved. For repetition and clarification, reference is made to modules 3 and 4. If the pilot is in a single-leg stance phase during a step, special attention must be paid to maintaining balance. Before lowering the pilot, balance must be completely restored. In such cases, performing the emergency procedure without a seating aid is recommended.

The correct application of procedural information requires specific prior knowledge. The corresponding sequence of actions for this obstacle is provided in the form of a HTA, which represents the prerequisite knowledge needed to apply the procedural instructions safely and accurately.

To facilitate understanding, detailed instructional videos are used to illustrate both the HTA and the corresponding sequence of correct movement execution for the obstacle. These videos must be viewed and internalized by the trainers without the pilot present to ensure safe and competent instruction.

The knowledge conveyed in the preceding sections is subsequently assessed through a quiz. In this assessment, trainers must arrange the HTA action steps in the correct order. The test is only considered passed if all questions are answered correctly. Only after successful completion is the next module section unlocked, in which the practical instruction of the *Moving Parcel* with the pilot takes place. This serves as a safety mechanism, ensuring that the trainer begins the practical training only after being fully prepared.

### Training execution with the pilot

#### Pre-training with the pilot

At the beginning of the preparation phase with the pilot, a detailed preparation checklist (Appendix A) is completed that covers both organizational and safety-related aspects. The checklist includes documentation of general session information such as the session number, date, role assignments and verification of the pilot’s clothing. It also contains preparation, including bladder emptying before training, preparation of the training team, including the presence of a same-gender person for skin inspection, and technical preparation of the exoskeleton, including confirmation of a full battery charge and the availability of all fixators and components. Furthermore, all required auxiliary equipment, such as a helmet, forearm crutches, and rope systems, is checked, and the training environment is inspected to ensure safety and completeness.

#### Training of the movement sequence

The module *Moving Parcel* is designed according to the principle of progressive assistance and consists of three consecutive levels of support. The aim of this structure is to enable a gradual reduction of external guidance and thereby promote increasing automation of skills on the part of the trainers. In addition, all obstacles are divided into part-task exercises, as they represent safety-critical skills that require a high degree of automation.

In *Level 1*, the complete HTA is provided and divided into part-task exercises, ensuring that the trainer has access to the entire sequence of actions in detail while guiding the movement. Table 5 presents the HTA for *Moving Parcel* in level 1, divided into three part-task exercises that are practiced sequentially in a progressive chain. First, part-task 1 is trained in isolation with full assistance and subsequently evaluated. Afterwards, part-tasks 1 and 2 are practiced in combination, then evaluated and followed by the complete movement sequence. Upon successful completion of the obstacle at a given level, the subsequent level can be entered.

**Table 5:** Hierarchical task analysis *Moving Parcel* in level 1

Level 1: moving parcel	
Part-task 1: approach and pick up the parcel	
1.1	Instruct the pilot to walk toward the parcel using double steps
1.2	Instruct the pilot to take a stable position in front of the box (table height approx. 75 cm)
1.3	Let the pilot shift their weight slightly forward
1.4	Ask the pilot to place the forearm crutches securely so that they do not tip over
1.5	Let the pilot grasp and lift the parcel with both hands
1.6	Instruct the pilot to place the parcel into the prepared bag
1.7	Let the pilot position the bag securely to ensure safe walking (e.g. hang around the handles of the bag)
1.8	Instruct the pilot to correctly grasp the forearm crutches again and return to the neutral standing position
Part-task 2: turning and walking with the parcel	
2.1	Instruct the pilot to initiate a turn to move around the table
2.2	Instruct to repeat the movement until the desired position is reached
2.3	Instruct the pilot to continue walking with double steps while securely carrying the bag
Part-task 3: placing the parcel and finish	
3.1	Let the pilot assume a neutral standing position
3.2	Instruct the pilot to place the forearm crutches securely so that they do not tip over
3.3	Instruct the pilot to open the bag and remove the parcel
3.4	Let the pilot place the parcel in the designated drop-off area
3.5	Instruct the pilot to correctly grasp the forearm crutches again and return to the neutral standing position
3.6	Instruct the pilot to continue walking with double steps until the finish line of the task is reached

In *Level 2*, the provided guidance is reduced to key terms that represent the essential action steps of the HTA, as shown in Table 6. This reduction encourages the trainer to recall the correct movement instructions increasingly from memory. At this stage, the obstacle is practiced only as a complete sequence, since performing level 2 requires the successful completion of level 1.

**Table 6:** Hierarchical task analysis *Moving Parcel* in level 2

Level 2: moving parcel	
1. Double steps towards the parcel	
1.1	Walk to the parcel with double steps
2. Pick up the parcel	
2.1	Assume a stable position in front of the box
2.2	Shift your weight slightly forwards
2.3	Put down forearm crutches safely
2.4	Lift the parcel with both hands
3. Pack the parcel into the bag	
3.1	Place the parcel into the bag
3.2	Position the bag stably
3.3	Grasp the forearm crutches and assume a neutral standing position
4. Turning	
4.1	Initiate rotation to move around table
4.2	Repeat the movement until the desired position is reached
5. Walk with parcel	
5.1	Continue with double steps, carry bag safely
6. Put down parcel	
6.1	Assume a neutral standing position
6.2	Put down forearm crutches safely
6.3	Open the bag and remove the parcel
6.4	Place the parcel in the storage position
6.5	Grasp the forearm crutches and assume a neutral standing position
7. Completion	
7.1	Walk to the end of the task in double steps

In *Level 3*, the trainer receives only brief prompts as support, as shown in Table 7. This final level is designed to promote independent and fully automated execution of the training instruction. Trainers are expected to guide the movement sequence autonomously and accurately.

**Table 7:** Hierachical task analysis *Moving Parcel* in level 3

Level 3: moving parcel	
1	With double steps to the table with parcel
2	Lift parcel and place in bag
3	Turn and walk past the table
4	Continue with double steps, carry bag
5	At second table: remove parcel, place on table
6	Finish the task with double steps

#### Post-training with the pilot

After completion of the practical exercises, the follow-up checklist (Appendix B) is filled out. This checklist includes both general information such as session number, date, and role assignments, as well as qualitative and quantitative evaluation data. The trainer documents feedback provided by the pilot, the trainers, and the entire team. In addition, video and protocol analyzes are conducted, standing and walking times are recorded, and potential pressure marks identified during the skin inspection are documented. Subsequently, the performance is assessed using the evaluation schemes described previously. Furthermore, the pilot completes the Borg scale [35] for perceived exertion and a subjective questionnaire.

#### Evaluation schemes and definition of done

The assessment of training performance represents a central component of the training concept and is closely linked to the learning progress of all participants involved. Two specific evaluation schemes were developed for this purpose: one for the trainers and one for the pilots. The evaluation scheme for the trainers is completed by the pilots, while the evaluation scheme for the pilots is completed by the trainers. This reciprocal assessment ensures that each exercise within a training session is evaluated by both parties. Such a bidirectional evaluation structure enables an objective measurement of performance levels and ensures transparent documentation of progress on both sides. Tables 8 and 9 show the definitions of the individual valuation levels for both sides.

**Table 8:** Evaluation scheme for pilot (to be assessed by the training lead)

Value	Meaning	Description
5	No assist	Task is performed safely and independently under supervision, without any physical assistance (movement may be accompanied by holding bars, but without any applied force)
4	Minimal assist	Task is performed with minimal assistance, but mostly safely and correctly
3	Moderate assist	Task can only be performed with noticeable assistance. Execution is partly unsafe or incorrect
2	Close contact guard	Task can only be performed with substantial assistance. Execution is unsafe and unstable
1	Total assist	Task can only be performed with full assistance or not at all

**Table 9:** Evaluation scheme for training lead (to be assessed by pilot)

Value	Meaning	Description
5	Correct, safe and competent instruction	The trainer provides correct, safe, and competent guidance. Instructions are clear, precise, and tailored to the needs of the participants
4	Correct instruction with minimal uncertainty	The trainer gives correct, mostly precise, and effective instructions with minimal ambiguity or uncertainty
3	Correct instruction with occasional uncertainty	The trainer gives correct instructions, but occasional uncertainty or lack of clarity occurs
2	Correct instruction with major uncertainty	The trainer shows significant uncertainty during instruction. The instructions are correct but unclear or unfocused
1	Incorrect instruction	The trainer provides incorrect or faulty guidance

A training session is considered successfully completed if the following definition of done is met:

*(1) each exercise at the respective level has been rated with at least four out of five points by both the pilot and the trainer; (2) the Borg scale shows a maximum value of 14; and (3) the subjective questionnaire has been completed in full.*

Only when these conditions are satisfied may the next training session proceed. Progress within the training program is always determined by the lower of the two evaluations provided independently by the pilot and the trainer. If either one of them achieve less than 4 out of 5 points in all exercises of a session, the entire session must be repeated in its original form. In contrast, if only certain exercises or a single exercise within a level are not passed, only those specific exercises within the same level are to be repeated in the subsequent session. The valuation key for this is shown in Table 10.

**Table 10:** Valuation key and progression rules

Rating	Meaning/consequence
4 or 5	Task passed—no repetition required
3	Task not passed—must be repeated in the *current level* until both pilot and trainer achieve at least a 4 or 5
Below 3	Task not passed—must be repeated in the *next lower level*

The lowest rating counts and determines the next step. Once a task in level 3 has been rated at least 4 or 5 by both sides, it does not need to be evaluated again

### Intermediate training sessions and final rehearsal

To consolidate and integrate the acquired movement sequences, intermediate training sessions are scheduled throughout the program. The first intermediate session takes place after the completion of the fundamental movement exercises and includes the standardized 10-Meter Walk Test [34]. During this session, a choreographed sequence of previously learned movements, such as standing up, walking, and turning, is performed in a fixed order.

In the second half of the training plan, within the obstacle-specific training phase, three additional intermediate sessions are conducted. In these sessions, all obstacles trained up to that point are completed in the official order of the CYBATHLON competition. The final session 38 serves as a full-scale rehearsal: all obstacles are performed under realistic competition conditions within a ten-minute time limit. This session may be repeated multiple times until all participants consider themselves sufficiently prepared for the competition.

## Discussion

The presented work pursued the goal of developing a methodology for a scientifically grounded and individualizable training concept for exoskeleton training that meets the specific requirements of CYBATHLON 2024, as well as the individual requirements of trainers and pilots. The methodology was developed in response to a concrete practical need for such a systematically designed framework. Existing training approaches lacked systematic structure and relied heavily on individual team experience, while no adaptable methodologies addressing these requirements could be identified in practice or literature. The focus was on the qualification of trainers, who are to be enabled to prepare pilots specifically for the competition with the help of a training program. The resulting concept therefore constitutes a practical tool that both meets current requirements and provides a basis to prepare for future competitions.

A key strength of the developed approach lies in its integrative and dynamic structure. The methodology for creating the training plan includes a temporal component that enables training to be planned and adjusted over time. In addition, the development of training materials is embedded within the process, resulting in practical and ready-to-use resources. However, a limitation of the current work lies in the limited testing and validation of the program in practice, as well as its relation to the EXO I system. The device-specific constraints of this model defined in “Background analysis of relevant information” section shaped the resulting training concept by excluding obstacles that could not be physically accomplished with this system. When applying the methodology, training design should account for the specific limitations of the respective device to ensure appropriate adaptation to each use case. Despite this, the overall methodological framework provides universally applicable guidance and is not restricted to particular device types, maintaining flexibility through the use of templates and adaptable modules for different pilots, teams, exoskeletons and future competitions.

Furthermore, the development process was guided by a structured procedure for literature and context analysis that integrated multiple perspectives. Conducting an interview study made it possible to identify key requirements for an exoskeleton training program specifically designed to prepare participants for the CYBATHLON competition. These requirements were essential for achieving the objectives of this work. Through expert interviews, a range of relevant information was collected, and several categories were identified as crucial for exoskeleton training. Although the sample size of five experts was relatively small, it included a broad spectrum of stakeholders from both exoskeleton industry and the CYBATHLON community. Regarding the qualitative content analysis, no weighting was applied to the identified categories and all aspects were treated equally. Introducing a prioritization based on frequency or perceived relevance could add analytical depth and help future studies identify less relevant elements within the training concept.

The explicit adaptation of the 4C/ID model to the CYBATHLON context represents a novel contribution, as no existing methodology in the literature currently addresses the systematic design of training concepts for exoskeleton training. The literature analysis confirmed the limited number of studies focusing on exoskeleton training design. Finally, by focusing on trainer enablement, the concept introduces a new approach that recognizes the essential interdependence between pilot and trainer. Since pilots cannot train effectively on their own, the empowerment of trainers become a decisive factor for performance outcomes.

It should be noted that the 4C/ID model does not represent the developed training concept in a temporally anchored format. Therefore, it cannot illustrate that different learning tasks are combined within a single session to promote variability, nor that previously performed movements are reintegrated in later sessions to, for example, refresh and stabilize critical skills. However, when examining the resulting training plan, it becomes evident that the training was not designed to practice a single movement exclusively through successive levels of difficulty, but rather to integrate multiple movements at different levels within one session in order to ensure a high degree of variability.

With regard to knowledge assessment, the training design entails certain limitations. Procedural knowledge is evaluated by increasing the difficulty of learning tasks while gradually reducing the level of instructional support provided to trainers. The evaluation of training outcomes achieved through the application of the training concept allows inferences about the trainers’ ability to convey procedural knowledge. However, the results may be influenced by individual differences among pilots, such as strength, mobility, or prior experience. Therefore, future research should include a systematic evaluation of the training in practice to examine these aspects in greater depth and to validate the effectiveness and adaptability of the developed methodology under real-world conditions.

Finally, it is important to emphasize the significant impact of individual differences among pilots. Factors such as the level of spinal cord injury or physical attributes like strength and mobility can strongly influence training needs, learning outcomes, and safety, thereby increasing the demands placed on trainers. The developed training concept, however, is designed to accommodate such individual variability through its flexible and modular structure. If appropriately applied by the trainer, the concept allows for tailored adaptations within each session to meet the specific requirements of individual pilots.

## Conclusions

Overall, the developed methodological framework can be utilized to develop practical training concepts for various exoskeleton systems. The training concept presented in this work, implemented as an e-learning platform based on this framework, demonstrates its applicability using the RISE EXO I as an example case. This concrete application can provide significant value for different exoskeleton systems in preparation for CYBATHLON 2028 or other comparable competitions, as the training was specifically designed around CYBATHLON requirements.

Analyzes of previous CYBATHLON events indicate that core task structures have remained largely consistent over several years, with only partial adjustments and occasional replacement of individual obstacles within the EXO discipline. Nevertheless, before each new training cycle, a thorough review of current regulations and obstacle specifications is required to implement necessary updates within the training system. Exoskeleton systems sharing similar device-specific constraints with the exemplary RISE device are more likely to adapt selected intermediate results of the developed training concept.

Furthermore, it is essential to evaluate the theoretical training concept in practical settings and, if necessary, introduce modifications to improve its applicability in real training contexts. A conceivable future direction could also involve formally defining the numerical effects of different exoskeleton types with different device-specific constraints on training requirements and task performance, which could facilitate the application of automated planning and scheduling methods to create individualized training plans. For safety-related modules and fundamental movement patterns, only minor adjustments are expected, primarily depending on the technical development of future exoskeleton models. Importantly, by enabling individualized adaptations and systematic trainer qualification, the framework offers a transferable methodology that can serve as a model for other exoskeleton systems still in research or development. This transferability underscores the broader relevance of the work and highlights its contribution to promoting effective and user-centered training practices.

In conclusion, the developed methodological framework provides a robust foundation for continuous advancement and for targeted preparation for future CYBATHLON competitions and real-life applications alike.

## Figures and Tables

**Fig. 9:**
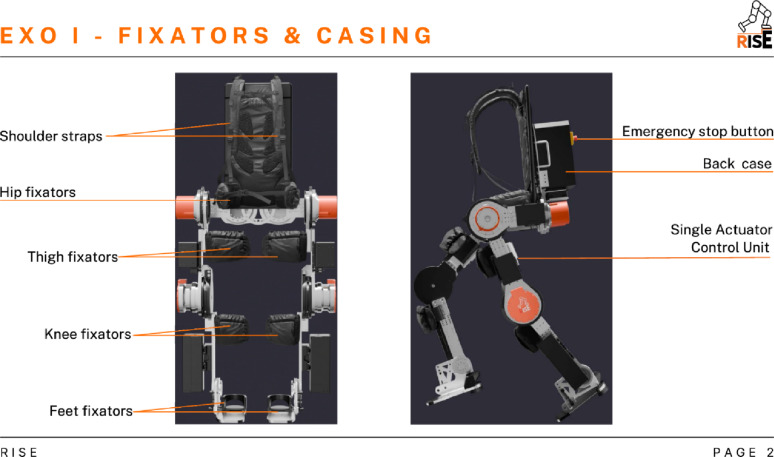
Representative expert from the preparation materials for EXO I

**Fig. 10:**
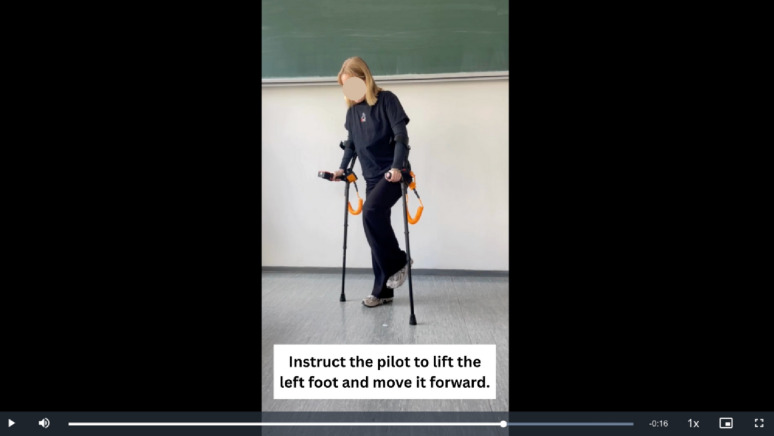
Screenshot from the video of the double step

## Data Availability

Interview transcripts are not available for reasons of confidentiality. The online course materials can be made available upon request.

## Appendices

### Appendix E: Representative excerpt from the preparation materials for EXO I

See Fig. 9.

### Appendix F: Screenshot from the video of the double step

See Fig. 10.

## Abbreviations

SCI: Spinal cord injury
6MWT: 6-Minute Walk Test
10MWT: 10-Meter Walk Test
4C/ID: Four-component instructional design
non-rec: Non-recurrent
rec: Recurrent
HTA: Hierarchical task analysis
